# Bladder and bowel dysfunction in Down syndrome with neural tube defect: case report and review of the literature

**DOI:** 10.1186/s13052-023-01412-z

**Published:** 2023-07-20

**Authors:** Roberta Onesimo, Cristiana Agazzi, Luca Massimi, Valentina Giorgio, Chiara Leoni, Giuseppe Zampino, Claudia Rendeli

**Affiliations:** 1 Rare Diseases Unit, Fondazione Policlinico Universitario Agostino Gemelli -IRCCS, Largo Agostino Gemelli 8, Rome, Italy; 2 Pediatric Unit, Fondazione Policlinico Universitario Agostino Gemelli -IRCCS, Rome, Italy; 3 Neurosurgery Unit - Fondazione Policlinico Universitario Agostino Gemelli -IRCCS, Rome, Italy; 4 Università Cattolica del Sacro Cuore, Rome, Italy; 5 Spina Bifida Center - Fondazione Policlinico Universitario Agostino Gemelli -IRCCS, Rome, Italy

**Keywords:** Down syndrome, Neural tube defect, Bowel bladder dysfunction, Pelvic floor rehabilitation, Case report

## Abstract

**Background:**

Down syndrome is a genetic disorder caused by trisomy of chromosome 21 and characterized by an increased risk of multiorgan involvement. In Down syndrome children, functional constipation and lower urinary tract infections have been described, together with higher risk for incontinence and delayed sphincter control. At present, to our knowledge, no clear association between Down syndrome, Bladder Bowel Dysfunction and neural tube defects has been previously described.

**Case presentation:**

We describe two female patients with Down syndrome presenting Bladder Bowel Dysfunction in association with neural tube defects, who both underwent personalized multidisciplinary intervention and pelvic floor rehabilitation, with good clinical outcomes.

**Conclusion:**

At present, no screening program has been established in order to rule out neural tube defects or neurogenic urinary anomalies in Down syndrome patients presenting bowel and/or bladder dysfunction. In our opinion, presence of spinal abnormalities, despite rare, may be contribute to urinary symptoms and should be ruled out in patients presenting progressive or persistent Bladder Bowel Dysfunction. Early diagnosis and management of spinal cord defects associated with neurogenic urinary dysfunction may allow to prevent possible complications.

## Background

Down syndrome (DS) is the most common chromosomal abnormality in humans [1]. DS is caused by trisomy of chromosome 21 and multiple health issues have been described, mostly involving the nervous, cardiovascular and musculoskeletal systems [2].

The discovery of a link between a supernumerary chromosome 21 and DS was first described in 1959 [3], and in 1960 Berg et al. described the first cases of DS with renal and urological malformations [4]. In the following years, a variety of urological abnormalities have been described in DS [5], with a relatively high incidence ranging from 3.5 to 21.4% [6, 7].

Life expectancy of DS individuals has increased dramatically during the last decades [8, 9]. Recently, it was shown that DS patients can develop renal disorders, voiding problems and urinary incontinence [10]. It is well known that DS patients are at risk for infections, but higher incidence of urinary tract infections in respect to the general population was never proven [11]. Combined intestinal and urinary tract dysfunction are common in DS; indeed, constipation and/or encopresis associated with severe disorders of urinary tract and fecal elimination are well established in DS [12].

At present, to our knowledge, no clear association between DS, Bladder Bowel Dysfunction (BBD) and neural tube defects (NTDs) has been described [13].

We describe two cases with DS and BBD presenting NTDs, both successfully treated with personalized approach.

## Case presentation

We describe two female pediatric patients with Down syndrome presenting Bladder Bowel Dysfunction in association with neural tube defects, who both underwent personalized multidisciplinary intervention and pelvic floor rehabilitation, with good clinical outcomes.

### Case 1

An 8-year-old female with DS and moderate degree of intellectual disability, regularly followed-up at the Congenital Defects and Rare Diseases Unit, was referred to the pediatric urologist for recurrent urinary tract infections. No skin markers of occult spinal dysraphism or peripheral neurologic involvement were found. Urodynamic studies showed an elevated vesical pressure together with defective detrusor functioning during filling phase, while cystography revealed an abnormal shaped bladder. The patient, therefore, received a diagnosis of detrusor sphincter dyssynergia.

**Fig. 1:**
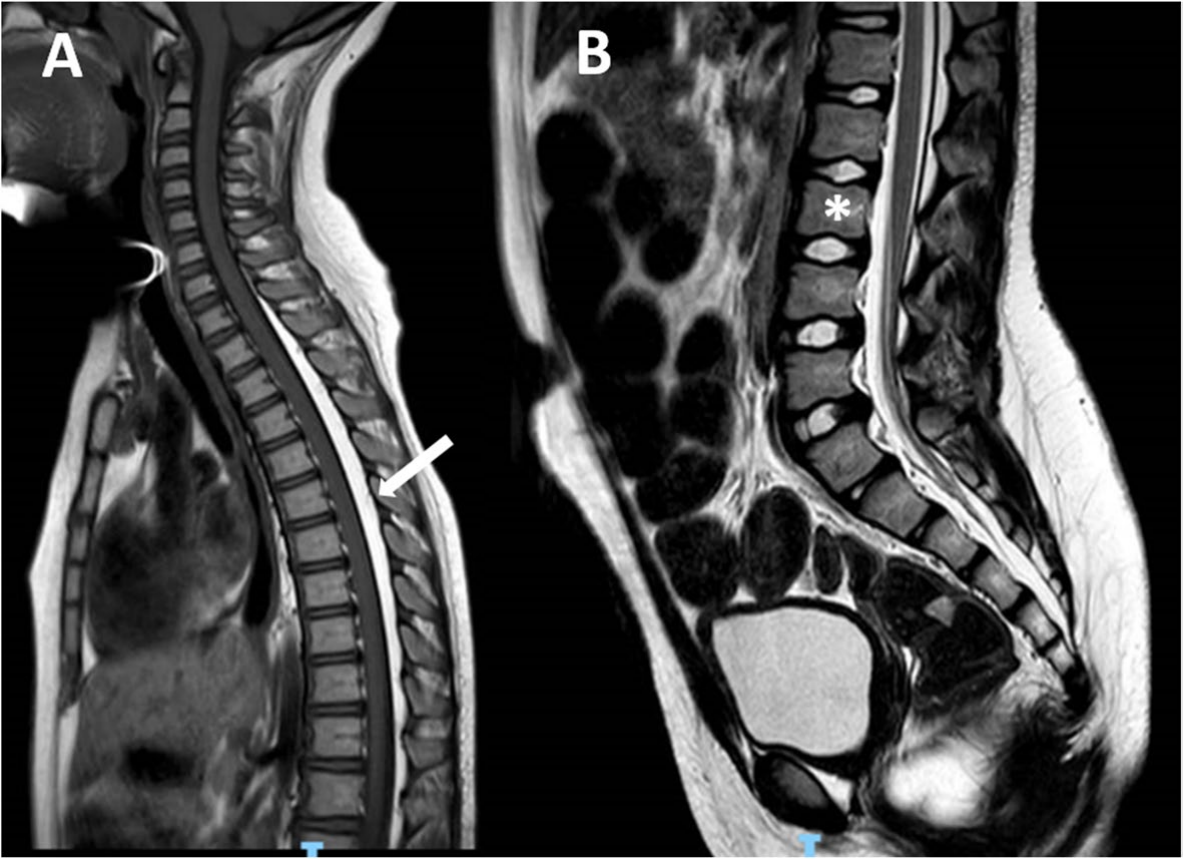
(case #1): **A** T1 sagittal view showing the epidural dorsal lipoma (arrow); **B** T2 sagittal view demonstrating the lower position of the conus (L3 level, asterisk) and the L5 sacralization

**Fig. 2:**
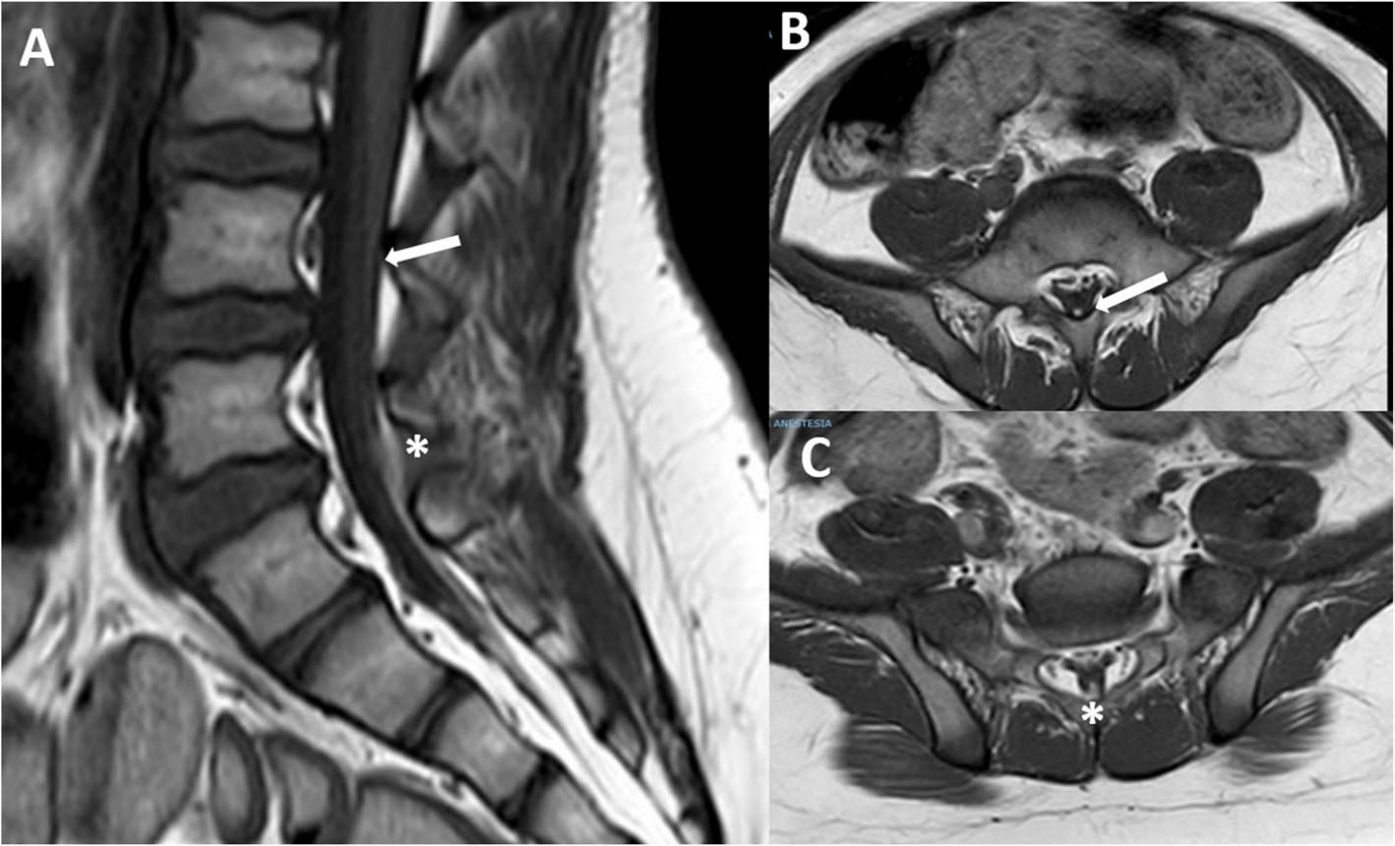
(case#1): the filar lipoma can be appreciated on T1 MRI. The upper portion is very thin (**A** and **B**, arrow) while the lower portion is a little larger (**A** and **C**, asterisk)

Later, the patient also complained of severe chronical constipation and incomplete emptying of the urinary bladder. Hence, diagnosis of BBD was postulated and spinal cord MRI was prescribed. Imaging studies showed the presence of an epidural dorsal lipoma involving the segments from T1 to L3 (Figs. 1 and 2). Finally, neurosurgical intervention involving laminectomy of L5 and resection of the filum terminale was performed.

Pelvic floor physical therapy was performed for a total of 2 months, resulting in improvement of constipation symptoms. Moreover, concerning urinary problems, no additional episodes of urinary tract infections occurred and complete urine continence was achieved.

### Case 2

Parents of an 8-year-old female affected by DS reported frequent episodes of enuresis and occasional daytime symptoms. Additionally, parents referred urinary urgency. The girl had previously undergone a brain MRI for recurrent episodes of epilepsy, which showed no structural anomalies. The patient was also affected by autism spectrum disorder (ASD), moderate-to-severe intellectual disability and celiac disease.

At physical examination, no skin markers of occult spinal dysraphism or peripheral neurologic involvement were detected.

Urodynamic studies revealed a low urinary flow rate in association to high detrusor pressure during the voiding phase, together with several uninhibited contractions. A large amount of postvoid residual urine was observed; hence, intermittent catheterization was prescribed.

**Fig. 3:**
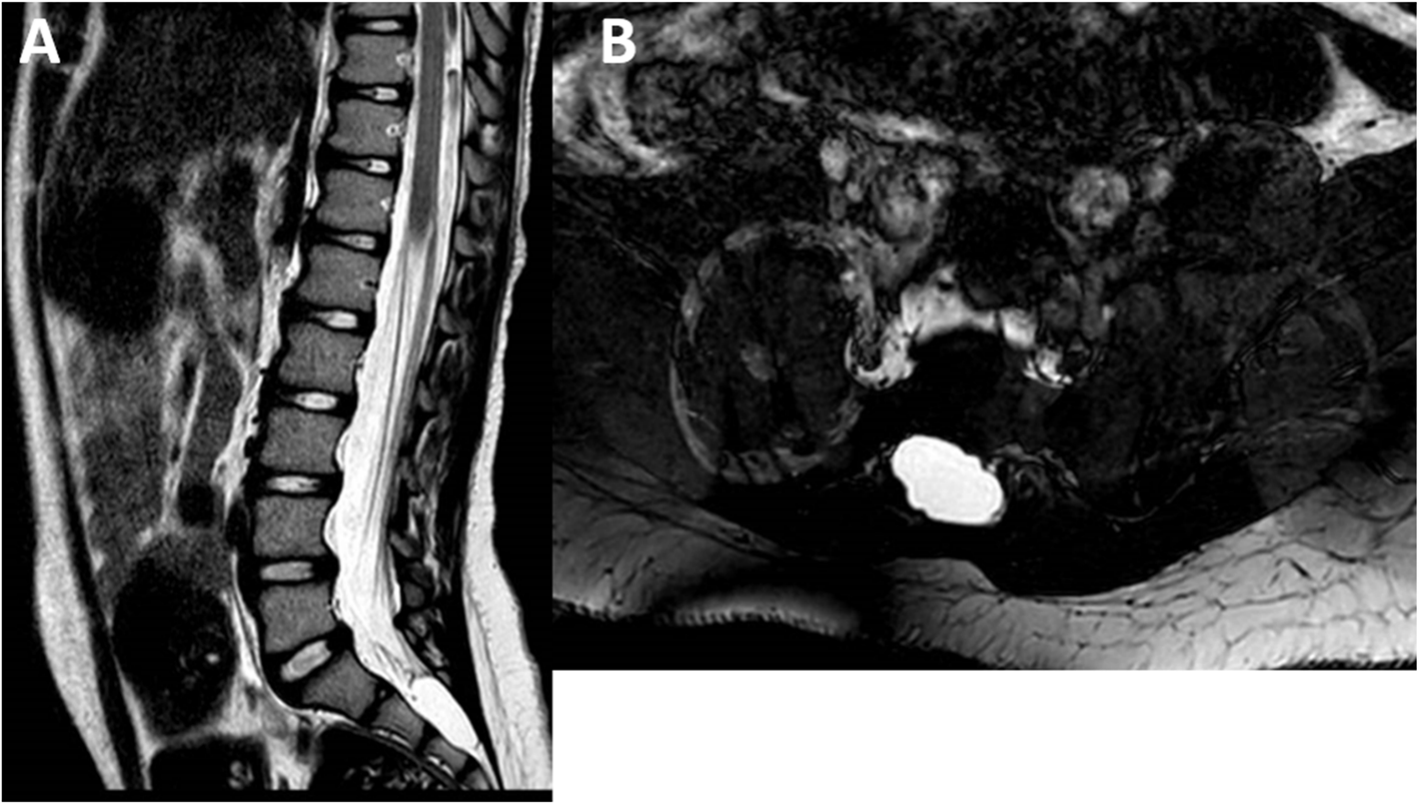
(case#2): **A** T2 sagittal MRI showing a dural megasac and an intrasacral cyst with upper displacement of the sacral roots; **B** Axial FIESTA MRI showing the right lateral extension of the cyst

Due to the persistence of urinary symptoms, spinal cord MRI was performed and revealed an intrasacral cyst, located caudally to an enlarged cul-de-sac, at the level of S2-S3 (Fig. 3). Such a lesion was considered as an intrasacral meningocele rather than a Tarlov cyst because of the missing involvement of the roots and the presence of a partial sacral schisis.

Pelvic floor muscle training was prescribed. Since enuresis resolved within 2 months of pelvic floor rehabilitation, no surgical intervention was required.

## Discussion and conclusion

It is well established that children with DS have an increased incidence of congenital nephro-urological anomalies [11, 12, 14]. Since bladder and bowel share the innervation from the same spinal district, urinary dysfunction is often accompanied by intestinal symptomatology. Indeed, BBD has been previously reported in DS [6, 12] with a clinical spectrum similar to our patients. Both patients in our study were females, in contrast to data reported in literature, where BBD was found to be twice more common in males than in females [12].

Functional constipation and lower urinary tract infections represent two conditions that can seriously affect quality of life in DS children [15]. Pelvic floor dysfunction is considered one of the major causes of BBD and was accordingly found in our two patients.

Kitamura et al. described lower urinary tract symptoms and abnormal urodynamic findings in DS children without UTI or severe constipation [6]. Additionally, Handel et al. described DS male children with and without neurogenic bladder [5]. Our study confirmed that uroflowmetry was safe and replicable in DS patients, also in presence of ASD.

DS is related to variable degree of intellectual disability which may mask specific signs of structural and/or functional defects responsible for urinary and fecal sphincter incontinence and enuresis persistence.

Severe intellectual disability has been associated with voiding dysfunction, and urinary continence is typically delayed in DS [16]. In the past, Kupferman et al. suggested that screening of kidneys and urinary tract should be part of the initial evaluation of every newborn with DS; moreover, he suggested adding an assessment of voiding function to check-ups for people with DS from the age of five [17].

Accordingly, in patients with DS, moderate-to-severe cognitive impairment and/or the presence of ASD, we suggest a multidisciplinary assessment, including pelvic floor evaluation, and acquisition of objective data by urodynamic and spinal MRI. Since incontinence is present in 64.0% of young children with DS, but only in 10–13% of teens and young adults [18], we suggest that the best age range to prescribe a spinal cord MRI may be preadolescence (9–12 years).

In conclusion, beyond focusing on urological/bowel symptoms, the presence of a spinal abnormality, contributing to the symptoms, despite rare, should be excluded in trisomy 21 patients with persistent BBD. Moreover, further investigations should be performed especially in presence of moderate-to-severe cognitive impairment and/or the presence of autism spectrum disorder in order to rule out NTDs.

Rehabilitative training should be offered to every patient with DS presenting BBD and should be carried out by experts in pediatric disability. Language impairment, often found in children with DS, may present a potential limitation in rehabilitation success and may be overcome by using appropriate communication methods as augmentative and alternative communication.

Implementation of DS guidelines in the field of BBD would allow to improve medical management, familial support and home environment, education, and rehabilitative training in DS children, so as to create a personalized rehabilitative treatment plan for each patient to promote health, functioning level and quality of life of children and adolescents with DS.

## Data Availability

The data that support the findings of this study are available on request from the corresponding author, [RO].

## Abbreviations

DS: Down Syndrome
NTD: Neural tube defect
BBD: Bladder Bowel Dysfunction
ASD: Autism spectrum disorder
